# The New York Dental Recorder

**Published:** 1849-10

**Authors:** 


### THE NEW YORK DENTAL RECORDER.

This valuable monthly makes its appearance on our table with great regularity, and we are always pleased to see it. A politician, without the latest news, would well be considered a fit emigrant for the upper fork of Salt river; and a Dentist *now*, without his periodical, embracing all that is new and valuable in the profession, might well be considered like some of the old mile stones on the National Road, which have stood so long you cannot read them. They are fine as mementos of the past, but are not really much needed since the introduction of Rail Roads. We believe in progress in science, but have very little faith in those who have progressed *so far;* that they have no need of any of the aids they might receive from those who are making researches in the same field.

We recently had a visit from a student, who came to our city to get instruments and a few items in practice. He thought his preceptor a *mighty* smart man. We asked him what periodicals he took; he said he really forgot. We asked if it was the Journal, the Recorder, the News Letter, or Register? No! but he thought it was a book about the size of Robinson Crusoe, and that he only had one number. We placed this man on the old turnpike, hard at work on a new stone to take his place—strange it the Rail Road does not run smash over his sign post.

But we hope the editor of the Recorder will excuse this digression, for we commenced this article intending to say that the October No. contains a new and important discovery in Dentistry, by C. S. Putnam, which we have reason to know is not very *new*. In our next we will give our reasons.

Also the proceedings of the New York Society of Dental Surgeons, which are of a very interesting character, and contain much that is practical and instructive, and a report of which we intend giving somewhat in full in our next.

The Recorder has now commenced its fourth volume, much improved in its appearances, with new type, and altered in size so as to afford two or three more pages of matter.

We hope its circulation may also be much increased.—[J. T.

